# Pan-cancer analyses of pyroptosis with functional implications for prognosis and immunotherapy in cancer

**DOI:** 10.1186/s12967-022-03313-x

**Published:** 2022-03-04

**Authors:** Aibin Liu, Lin Shen, Na Li, Liangfang Shen, Zhanzhan Li

**Affiliations:** 1 Department of Geriatrics, Xiangya Hospital, Central South University, Changsha, 410008 Hunan People’s Republic of China; 2 Department of Oncology, Xiangya Hospital, Central South University, No. 87, Xiangya Road, Kaifu District, Changsha, 410008 Hunan People’s Republic of China; 3 National Clinical Research Center for Geriatric Disorders, Xiangya Hospital, Central South University, Changsha, 410008 Hunan People’s Republic of China

**Keywords:** Pan-cancer, Pyroptosis, Immunotherapy, Tumor microenvironment

## Abstract

**Background:**

Programmed cell death is an active and orderly form of cell death regulated by intracellular genes that plays an important role in the normal occurrence and development of the immune system, and pyroptosis has been found to be involved in tumorigenesis and development. However, compressive analysis and biological regulation of pyroptosis genes are lacking in cancers.

**Methods:**

Using data from The Cancer Genome Atlas, we established a score level model to quantify the pyroptosis level in cancer. Multiomics bioinformatic analyses were performed to assess pyroptosis-related molecular features and the effect of pyroptosis on immunotherapy in cancer.

**Results:**

In the present study, we performed a comprehensive analysis of pyroptosis and its regulator genes in cancers. Most pyroptosis genes were aberrantly expressed in different types of cancer, attributed to the CAN frequency and differences in DNA methylation levels. We established a pyroptosis level model and found that pyroptosis had dual roles across cancers, while the pyroptosis levels were different among multiple cancers and were significantly associated with clinical prognosis. The dual role of pyroptosis was also shown to affect immunotherapeutic efficacy in several cancers. Multiple pyroptosis genes showed close associations with drug sensitivity across cancers and may be considered therapeutic targets in cancer.

**Conclusions:**

Our comprehensive analyses provide new insight into the functions of pyroptosis in the initiation, development, progression and treatment of cancers, suggesting corresponding prognostic and therapeutic utility.

**Supplementary Information:**

The online version contains supplementary material available at 10.1186/s12967-022-03313-x.

## Background

Programmed cell death is an active and orderly form of cell death regulated by intracellular genes that plays an important role in the normal occurrence and development of the immune system [1]. Pyroptosis is a new type of programmed cell death mediated by caspase-1, characterized by rapid rupture of the plasma membrane, followed by the release of cell contents and proinflammatory substances such as IL, thereby triggering an inflammatory cascade that leads to cell damage [2]. With the extensive development of research on pyroptosis, its complex biological functions are beginning to emerge. As a new type of programmed inflammatory necrosis, pyroptosis is involved in the occurrence and development of various diseases [3], including acute liver injury, rheumatoid arthritis, and acute kidney injury [4–6]. In recent years, pyroptosis has been found to be involved in tumorigenesis and development [7]. For example, human mesenchymal stem cells can induce pyroptosis by secreting interleukin-1β (IL-1β), which can lead to the death of breast cancer cells [8]. Euxanthin can significantly inhibit the proliferation, migration and invasion of hepatoma cells, and this process plays a main role in the mechanism by which euxanthin activates the pyroptosis signalling pathway mediated by Caspase-1 [9]. Simvastatin inhibits the proliferation and migration of non-small-cell lung carcinoma (NSCLC) cells. The mechanism may be related to the simvastatin-induced activation of the NLRP3 inflammatory body, caspase-1, IL-1β and interleukin-18 (IL-18), thereby inducing pyroapoptosis in NSCLC. Caspase-1 inhibitor attenuates the inhibitory effect of simvastatin on NSCLC [10]. Conversely, activation of the NLRP3 inflammasome promotes the proliferation and migration of lung adenocarcinoma A549 cells, which is related to the ability of the NLRP3 inflammasome to mediate the release of IL-18 and IL-1β through caspase-1-dependent or caspase-1-independent pathways [11]. Similarly, the NLRP1 inflammasome promotes melanoma growth by increasing Caspase-1 activity and promoting IL-1 β secretion [12]. In addition, pyroptosis is involved in the immune regulation process in tumours. Elion et al. explored the role of the RIG-I-mediated innate immune response in breast cancer cells, which was partly attributed to the activation of pyroptosis-inducing inflammatory cytokines [13]. Furthermore, Caspase 3/GSDME-dependent pyroptosis contributes to chemotherapy drug-induced nephrotoxicity. These results show that pyroptosis is involved in tumour progression and treatment, and better understanding the mechanism of pyroptosis will facilitate new approaches to tumour treatment.

In this study, we first performed a comprehensive analysis of molecular patterns, including somatic copy number alterations, mutations, deoxyribonucleic acid (DNA) methylation, pathway enrichment, the immune microenvironment, patient survival, effects on immunotherapy and drug resistance, across cancers. We also established a model of pyroptosis-related gene levels across 33 cancer types. Our results highlight the important role of pyroptosis in cancer and provide new insight into the functions of pyroptosis and therapy in cancer.

## Methods

### Datasets and source

We downloaded the following data from the University of California SANTA CRUZ (UCSC: https://xenabrowser.net/datapages/): marked copy number segment, DNA methylation (Illumina human methylation 450), gene expression RNAseq (HTSeq), somatic mutation (SNPx and small INDELs), and survival data. The lists of cancer types are presented in Additional file 1: Table S1. The drug response data and genomic markers of sensitivity were downloaded from the Genomics of Drug Sensitivity in Cancer (https://www.cancerrxgene.org/) and Cancer Therapeutics Response Portal (http://portals.broadinstitute.org/ctrp/) identifying and targeting cancer dependencies with small molecules. Immune-associated data, including immune cells and immunophenoscores, were downloaded from ImmuCellAI (Immune Cell Abundance Identifier) (http://bioinfo.life.hust.edu.cn/ImmuCellAI#!/). Three immunotherapy datasets (GSE13507: primary bladder cancer; GSE32894: urothelial carcinoma; GSE61676: non-squamous non-small cell lung cancer) were from the Group on Earth Observations (GEO) dataset (https://www.ncbi.nlm.nih.gov/gds). Thirty-three pyroptosis-related genes were obtained from a previous publication **(**Additional file 1: Table S2) [14].

### Differentially expressed gene analysis

To explore the expression differences in pyroptosis-related genes between cancerous and normal tissues, we performed differential expression analyses of 32 cancers using the “limma” R package [15]. A |Log2-fold change (FC)| value > 1 and adjusted *P* value < 0.05 were defined as threshold for indicating significant differential expression levels.

### Somatic copy number alteration and mutation analysis

Data on mutation, fusion, amplification, homozygous and heterozygous deletion and amplification were included to evaluate the copy number alterations and mutations of pyroptosis genes. We defined over five percent of the genes as having high-frequency copy number alterations. Pearson’s correlation coefficient was used to evaluate the association between copy number alterations and mRNA expression. The R “maftool” package was employed to evaluate the overall mutation of pyroptosis genes in cancers.

### DNA methylation analysis

DNA methylation (Illumina Human Methylation 450) data of 33 cancers were downloaded from the UCSC database. Some cancers do not have normal methylation data. The differential methylation of 14 cancerous tissues and normal tissues was determined using the Wilcoxon rank test. Genes were defined as hypomethylated or hypermethylated according to the adjusted *P* value (P < 0.05). The correlations between methylation and gene expression levels were evaluated using Spearman correlation. *P* < 0.05 was considered significant.

### Establishment of the pyroptosis level model

To evaluate the pyroptosis level in cancer, we calculated the pyroptosis score using single sample gene set enrichment analysis (ssGSEA) in the R ‘performed gene set variation analysis (GSVA)’ package [16]. The enrichment score was divided into positive and negative components. The normalized difference between positive and negative components was defined as the pyroptosis level. To evaluate the pathway enrichment of each sample, we performed GSVA and estimated the gene set enrichment of pyroptosis genes [17]. The Kyoto Encyclopedia of Genes and Genomes (KEGG) gene set (c2.cp.kegg. v6.2. symbols) was downloaded from the GSEA database (http://www.gsea-msigdb.org/gsea/index.jsp).

### Survival analysis

We evaluated the effect of pyroptosis levels on cancer survival prognosis. The following four survival outcomes were included: overall survival (OS), disease-specific survival (DSS), progression-free interval (PFI), and disease-free interval (DFI). We calculated the hazard ratio of the pyroptosis score in each cancer using Cox regression. The pyroptosis score was categorized into high and low groups according to the median. Kaplan–Meier analysis was used to compare the survival curves of the high and low pyroptosis groups. P < 0.05 was considered significant.

### Immune feature analysis

To investigate the association between pyroptosis and the immune microenvironment, we calculated the Pearson correlation coefficients between the pyroptosis score and immune parameters, including the immune score, stromal score, estimated score, and tumour purity.

Immune cells included B cells, T cells, myeloid dendritic cells, endothelial cells, NK cells, macrophages, haematopoietic stem cells, and immune cell subsets. Some immune-related pathways, matrix/metastasis-related pathways, and DNA damage repair pathways were also evaluated.

We also investigated the effect of the pyroptosis level on survival prognosis in three GEO datasets (GSE13507: primary bladder cancer, survival outcome: OS; GSE32894: urothelial carcinoma, survival outcome: PFS; GSE61676: non-squamous non-small cell lung cancer, survival outcome: OS).

### Drug sensitivity analysis and pyroptosis level

To evaluate the association between the pyroptosis level and small-molecule drugs, we calculated the Pearson correlation coefficients between the pyroptosis score and drug sensitivity percentage calculated by the percent viability curve approach. We identified and targeted cancer dependencies with small molecules using the Genomics of Drug Sensitivity in Cancer and Cancer Therapeutics Response portal dataset.

## Results

### Genetic alteration landscape of pyroptosis-related genes in cancer

**Fig.1:**
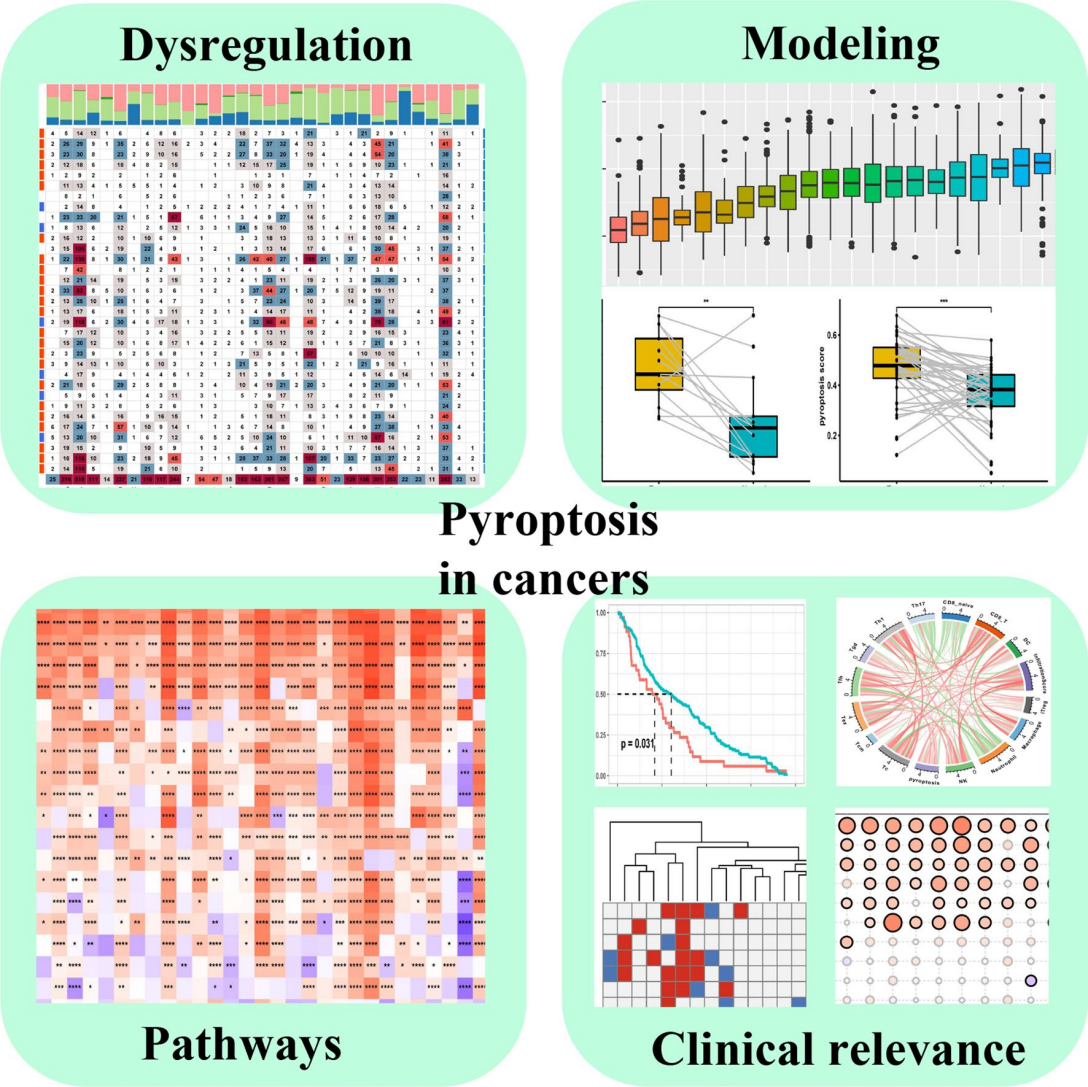
General overview of this study

**Fig.2:**
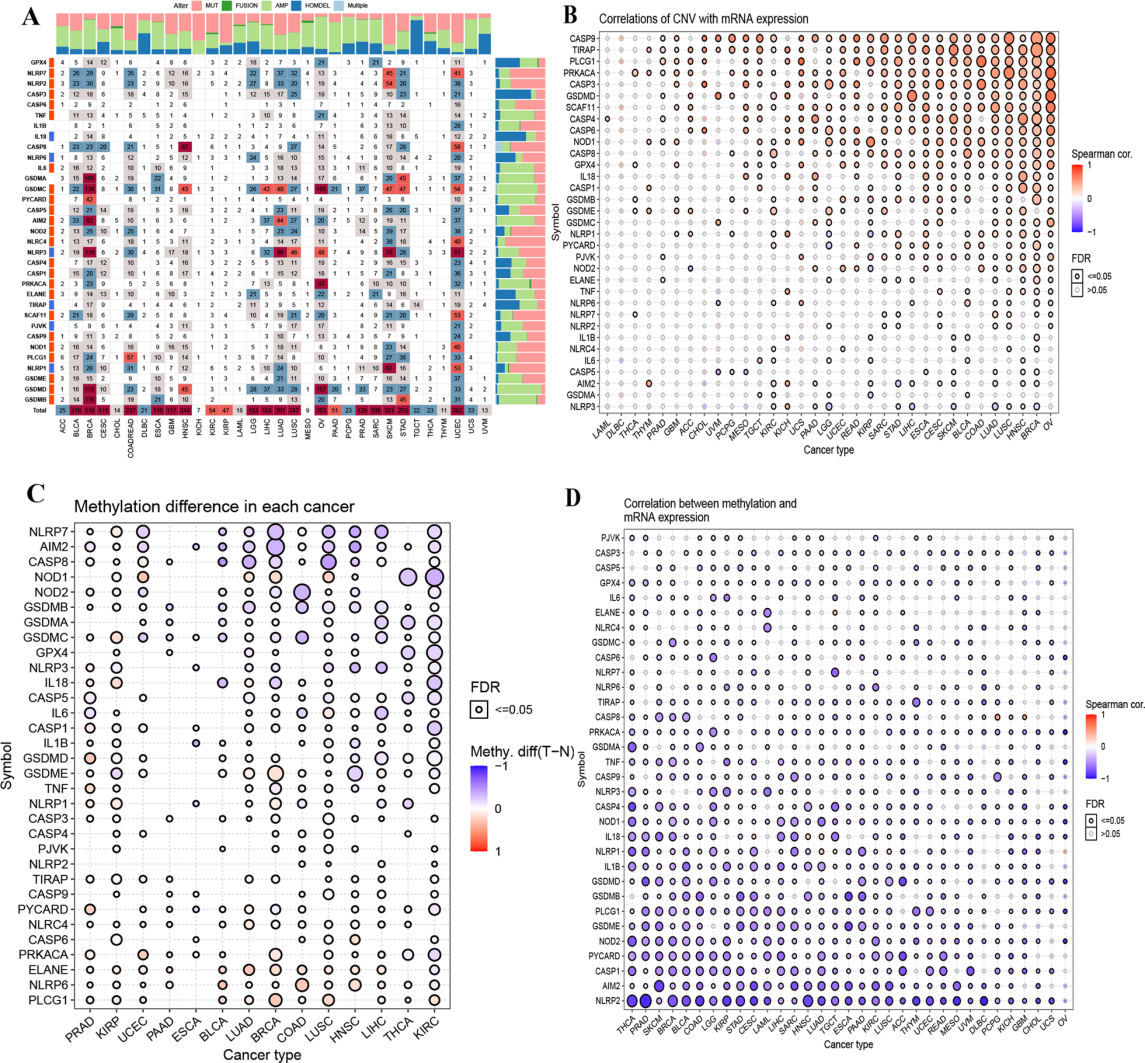
Genetic alteration landscape of pyroptosis-related genes in cancer. **A** Gene alterations in cancer. **B** Correlation of copy number variation and pyroptosis genes expression. **C** Methylation level differences in each cancer. **D** Correlations between methylation level and pyroptosis genes expression

In this study, we identified thirty-three genes that play critical roles in regulating pyroptosis by reviewing previous studies (Fig. 1). The lists of 33 pyroptosis-associated genes are provided in Additional file 1: Table S2. To determine the patterns of dysregulation of pyroptosis genes in cancer, we examined genomic data, including genetic variation, somatic copy number alteration, messenger ribose nucleic acid (mRNA) expression, and DNA methylation data of tumour and normal tissues from 32 cancer types. The overall alteration levels of pyroptosis genes ranged from 0.7% to 7.7%. Although the DNA alteration level was relatively low, 50% of tumours had at least one type of alteration (Fig. 2A). Mutations, amplifications and homozygous deletions accounted for the majority of pyroptosis gene alterations. Among these 33 genes, GSDMC showed the highest alteration frequency (7.7%), with amplification being the most frequent (5.5%).

The alteration rates of GSDMD and NLRP3 were similar (6.3% and 6.0%). The alteration rates of other genes ranged from 01 to 3%, and CASP6 had the lowest alteration level (0.7%). We further analysed the alteration patterns among all cancer types and found differences in alterations among different cancer types. UCEC showed alterations in all pyroptosis genes, while the alteration levels of all genes related to kidney chromophobe (KICH) and mesothelioma (MESO) were very low (Fig. 2A). Individual pyroptosis genes also showed differential amplification or deletion patterns among cancer types. Almost all cancers exhibited a high heterozygous amplification frequency, while the heterozygous amplification frequency was relatively low in thymoma (THYM), acute myeloid leukaemia (LAML) and thyroid carcinoma (THCA). The same trend was observed regarding heterozygous deletions in the three types of cancers. However, GSDMC and GSDMD had high homozygous amplification frequencies in ovarian serous cystadenocarcinoma (OV), oesophageal carcinoma (ESCA), uterine carcinosarcoma (UCS), breast invasive carcinoma (BRCA), stomach adenocarcinoma (STAD), liver hepatocellular carcinoma (LIHC), and uveal melanoma (UVM). The high homozygous deletion rates were scattered in most cancers (Additional file 2: Fig. S2A, B).

The gene mutations mainly consisted of missense mutations, nonstop mutations and multihit mutations. The mutation levels of these genes ranged from 0 to 3%. NLRP3 showed the highest mutation level (3%), and NLRP7, NLRP1, CASP8 and NLRC4 showed the same mutation frequencies (2%). The remaining genes had mutation frequencies of only 1% (Additional file 2: Fig. S2C). For cancer types, the mutation frequencies of pyroptosis genes are relatively low in all cancers and relatively high in skin cutaneous melanoma (SKCM). Furthermore, NLRP3, which encodes a pyrin-like protein containing a pyrin domain, functions as an upstream activator of nuclear factor (NF-kappa B) signalling and regulates inflammation, the immune response, and apoptosis, showed relatively high mutation frequencies in several cancers, including uterine corpus endometrial carcinoma (UCEC), SKCM, colon adenocarcinoma (COAD), LUAD, and lung squamous cell carcinoma (LUSC) (Additional file 2: Fig. S3). NLRP3 mutation was significantly related to the PFS, OS and DSS of UCEC patients. Other genes also showed significant associations with survival prognosis in multiple cancers (Additional file 1: Table S3).

### Aberrant expression of pyroptosis-related genes among cancers

We performed differential expression analysis of pyroptosis genes among cancers except MESO and UVM without normal tissues. Our results indicated that all pyroptosis genes were differentially expressed in at least one type of cancer. Some pyroptosis genes exhibited consistent expression patterns in multiple cancers. GSDMC, NLRP7, CASP5, PYCARD, IL18, IL1B and GSDMA were significantly upregulated in 22, 18, 18, 18, 18, 16 and 17 types of cancers, respectively (Additional file 2: Fig. S4). Protein kinase CAMP-activated catalytic subunit alpha (PRKACA), elastase neutrophil expressed (ELANE), NLRP1, pejvakin (PJVK), and CASP9 were significantly downregulated in 25, 24, 22, 25, and 23 types of cancers, respectively. Several pyroptosis genes showed cancer type-specific patterns. ELANE was significantly downregulated in almost all cancers but obviously upregulated in GBM (log2FC = 2.07) and LAML (FC = 12.15). CASP8 mutations are associated with increased risks of cancer, and low expression of CASP8 is closely associated with poor prognosis in patients with cancer; however, CASP8 expression was significantly upregulated in glioblastoma multiforme (GBM) and pancreatic adenocarcinoma (PAAD) [18]. IL6 seems to promote the development of cancer [19] and is significantly upregulated in lymphoid neoplasm diffuse large B-cell lymphoma (DLBC), GBM, PAAD, testicular germ cell tumours (TGCTs), and THYM but downregulated in adrenocortical carcinoma (ACC), BLCA, BRCA, kidney chromophobe (KICH), kidney renal clear cell carcinoma (KIRP), LAML, and LUAD. We also found that IL6 exhibited the opposite expression pattern in some subtypes of brain and kidney cancers. These results indicated that pyroptosis genes may differentially function in different cancers.

Copy number variation (CNV) is known to play a role in tumorigenesis development, and we further evaluated the association between CNV and pyroptosis gene expression. Pearson correlation analysis indicated that most pyroptosis genes were correlated with CNV in most cancers (Fig. 2B). For example, TIRAP, which is involved in the Toll-like receptor (TLR4) signalling immune system pathway, was significantly correlated with CNV in 29 types of cancers. PRKACA, which participates in cellular processes, including differentiation, proliferation, and apoptosis, was significantly associated with CNV in 27 cancers. These results showed that abnormal copy numbers of pyroptosis genes are common in most cancers and affect gene expression levels.

We also assessed the methylation levels of pyroptosis genes in tumour and normal tissues. We found that pyroptosis genes exhibited complex methylation patterns in the 14 types of cancers (Fig. 2C), and only ELANE showed hypermethylation in 12 types of cancers. We observed that NLRP7 (n = 8), AIM2 (n = 10), CASP8 (n = 6), GSDMA (n = 5), GSDMB (n = 8), and GSDMC (n = 12) mainly showed hypomethylation in most cancers, and PLCG1 (n = 11), NLRP6 (n = 13), ELANE (n = 11), CASP6 (n = 5), NLRC4 (n = 12), and PYCARD (n = 8) showed hypermethylation in most cancers. The methylation levels of pyroptosis genes differed significantly in 14 cancers (Additional file 1: Table S4). Spearman correlation analysis indicated a negative relationship between gene expression and overall methylation levels (Fig. 2D and Additional file 2: Table S5). These results suggested that DNA methylation regulates the expression of pyroptosis genes in cancers.

### Estimated modelling of pyroptosis levels and their association with prognosis among cancers

**Fig. 3:**
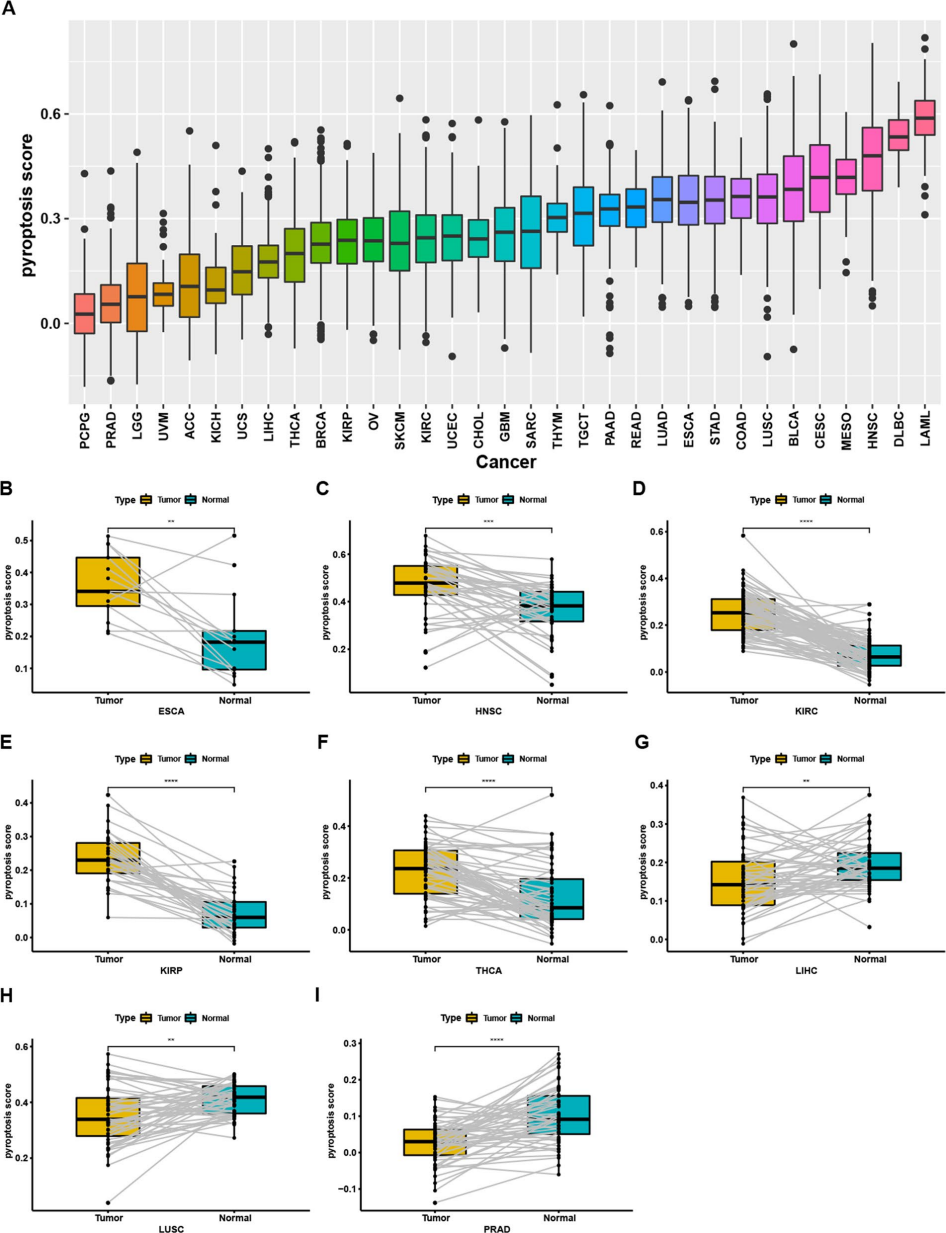
Establishing modelling of pyroptosis level in cancers. **A** Pyroptosis level in each cancer. **B–F** Elevated pyroptosis levels were found in ESCA, HNSC, KIRC, KIRP, and THCA. **G–I** Decreased pyroptosis leves were observed in LIHC, LUSC and PRAD

To further explore the role of pyroptosis in the development of tumours and understand pyroptosis-related biological processes, we built an estimated model of pyroptosis levels in all cancers based on enrichment scores by single-sample GSEA. We observed that LAML had the highest pyroptosis level, while pheochromocytoma and PGL had the lowest pyroptosis levels (Fig. 3A). We further compared the pyroptosis levels between tumours and normal tissues. We observed that the pyroptosis levels were significantly increased in oesophageal carcinoma (ESCA), head and neck squamous cell carcinoma (HNSC), KIRC, KIRP, and THCA (Fig. 3B–F), while the pyroptosis levels were significantly decreased in LIHC, LUSC and PRAD (Fig. 3G–I).

**Fig. 4:**
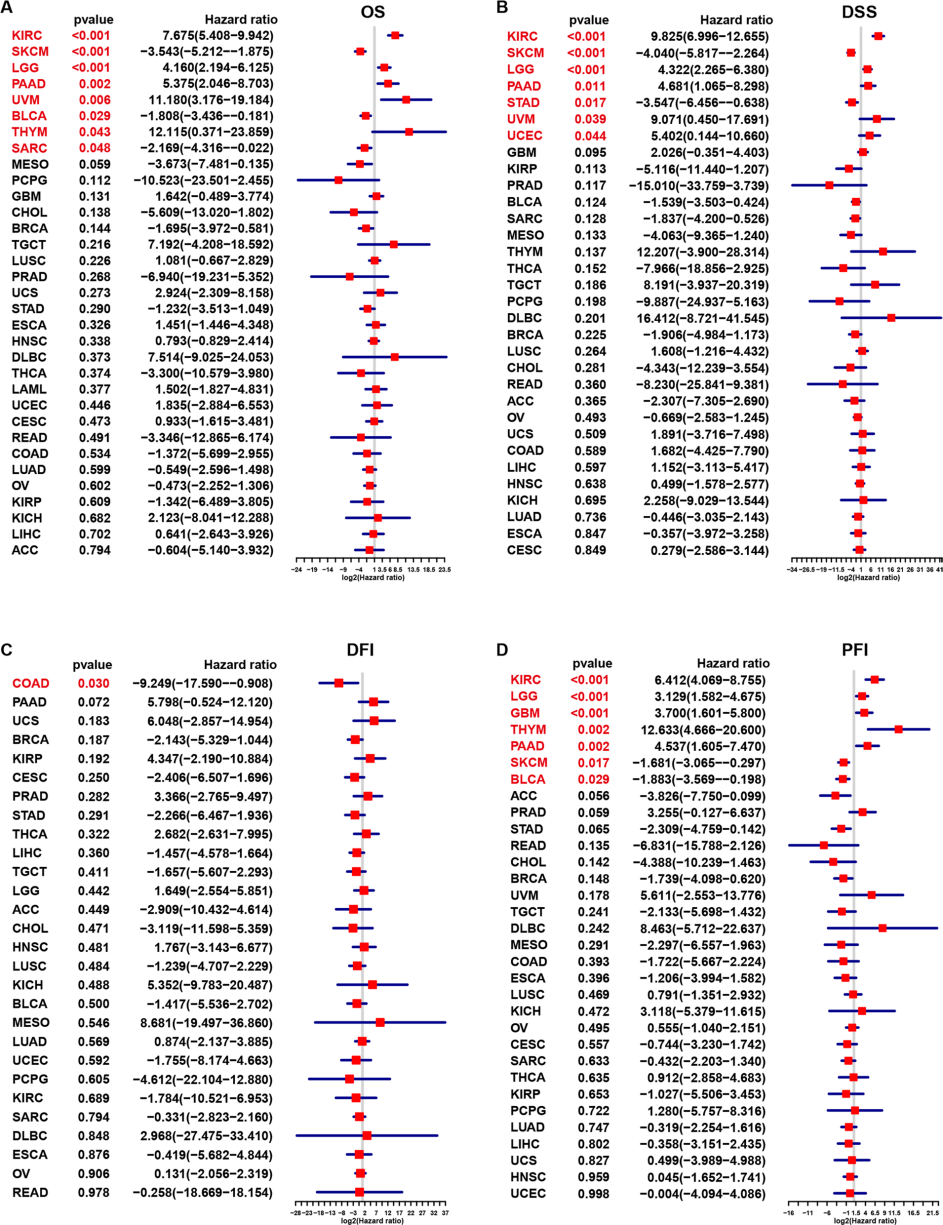
Correlations of pyroptosis level with prognosis in each cancer based on cox regression. **A** Overall survival. **B** Disease specific survival. **C** Progression free interval. **D** Disease-free interval

We further performed univariate Cox regression to evaluate the associations between pyroptosis levels and four survival outcomes, OS, DSS, DFI and PFI. The pyroptosis score was significantly associated with OS in eight types of cancer (Fig. 4A), KIRC (*P* < 0.001), SKCM (*P* < 0.001), LGG (*P* < 0.001), PAAD (*P* = 0.002), UVM (*P* = 0.006), BLCA (*P* = 0.029), THYM (*P* = 0.043), and SARC (*P* = 0.048), while the pyroptosis score was significantly associated with DSS in seven types of cancers (Fig. 4B), KIRC (P < 0.001), SKCM (P < 0.001), LGG (P < 0.001), PAAD (P = 0.011), UVM (P = 0.039), and UCEC (P = 0.044). However, the pyroptosis score was significantly associated with DFI in only one cancer (Fig. 4C), COAD (*P* = 0.030). A significant correlation between the pyroptosis score and PFI was also observed in seven cancers (Fig. 4D), KIRC (*P* < 0.001), LGG (*P* < 0.001), GBM (*P* < 0.001), THYM (*P* = 0.002), PAAD (*P* = 0.002), SKCM (*P* = 0.017), and BLCA (*P* = 0.029). Among these cancers, the elevated pyroptosis score was associated with poor survival outcomes for patients with twelve types of cancer (Additional file 2: Fig. S5A), ESCA, GBM, HNSC, KIRC, LAML, LGG, LUSC, PAAD, THYM, UCES, UCS, and UVM, while the elevated pyroptosis scores favoured survival for patients with eight types of cancer (Additional file 2: Fig. S5B), BRCA, KICH, MESO, SARC, SKCM, STAD, THCA and BLCA. The pyroptosis score had different clinical effects in some cancers. For example, pyroptosis was shown to be unfavourable for KIRC but favourable for KICH.

We also evaluated the associations between pyroptosis genes and tumour risks. Overall, the NLRP3, PJVK, TIRAP, IL18, NLRP1 and NLRP6 genes were thought to protect against cancer (Additional file 2: Fig. S6A, B), while the rest of the pyroptosis genes seemed to be correlated with cancer risk. Some genes showed different risk patterns. For example, IL6 is a risk gene in multiple cancers but plays a protective in only sarcoma (SARC). A similar result was also observed for PRKACA in several cancers. In contrast, TIRAP is a protective gene in KIRC, rectum adenocarcinoma (READ), and STAD but a risk gene in only BRCA. These results indicated that pyroptosis genes may play different roles in tumours.

### Pyroptosis-related pathways and immune signatures among cancers

**Fig.5:**
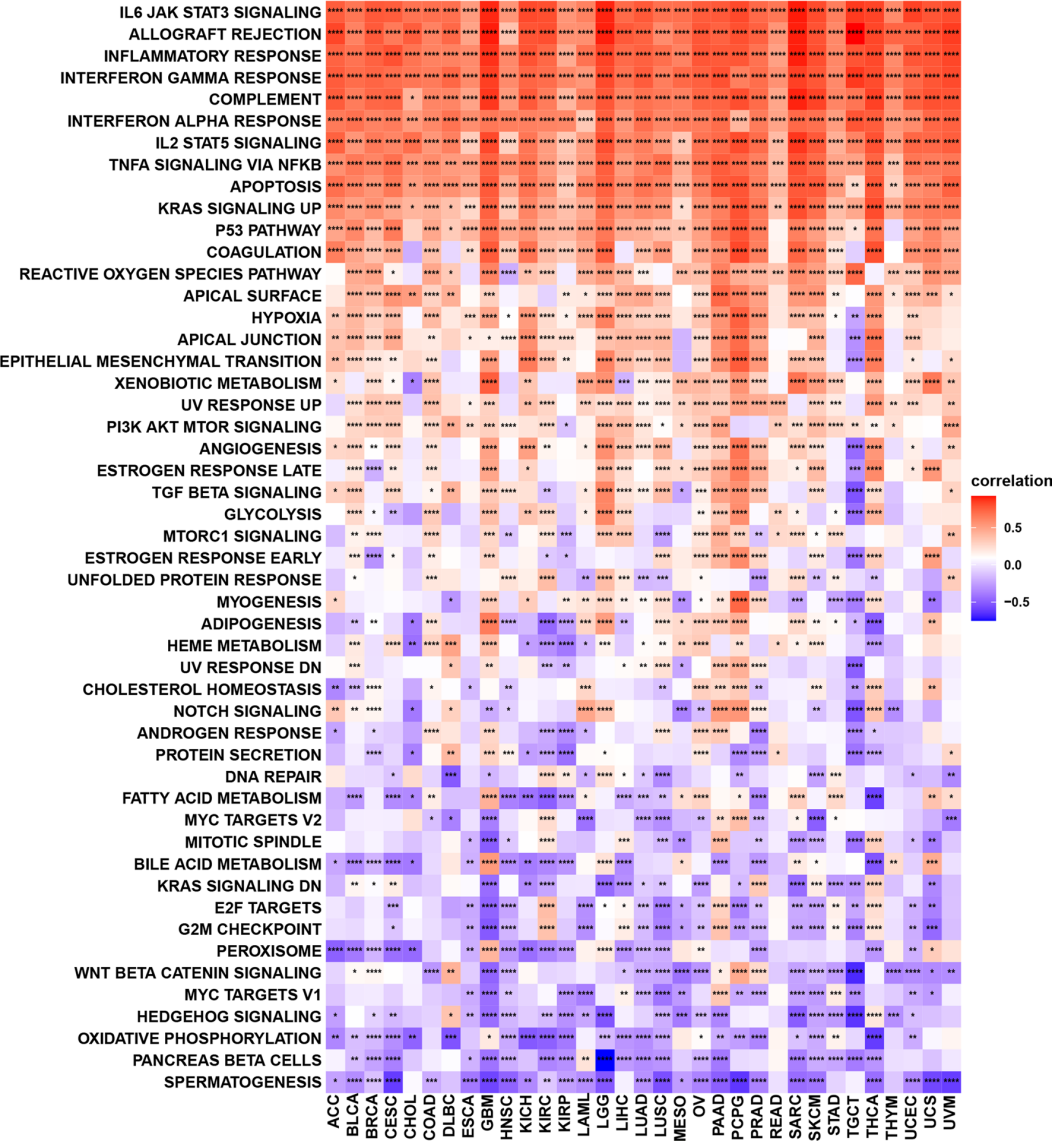
Gene set variation analysis for pyroptosis in cancers

To evaluate the associations between pyroptosis levels and pathways, we calculated Spearman correlation coefficients between pyroptosis scores and other genes and pathways using GSEA in all cancers. As shown in Fig. 5, IL-6/JAK/STAT3 signalling, allograft rejection, inflammatory response, IL2/STAT5 signalling, tumour necrosis factor (TNF-A) signalling via nuclear factor kappa B (NF-kB), apoptosis KRAS signalling and the P53 pathway were enriched in tumours with high pyroptosis levels, indicating that pyroptosis was positively associated with these pathways. Spermatogenesis (29 cancers), pancreatic beta cells (20 cancers), oxidative phosphorylation (24 cancers), hedgehog signalling (22 cancers), Wnt-beta catenin signalling (22 cancers), peroxisomes (20 cancers), and the G2/M checkpoint (22 cancers) were enriched in most tumours with low pyroptosis levels, which indicated that pyroptosis was negatively associated with these pathways. Other common pathways, such as the reactive oxygen species pathway, hypoxia, epithelial-mesenchymal transition (EMT), PI3K/Akt, and some metabolism-related pathways, were also enriched in multiple cancers. These pathways showed a positive association with the pyroptosis level. We further performed GSEA of six cancers (significantly associated with the pyroptosis level) based on significant survival analysis (Additional file 2: Fig. S7A–F). We observed that multiple immune-related pathways, such as the innate immune system, cytokine signalling in the immune system, and the adaptive immune system, were enriched in BRCA, KIRC, LUSC and PAAD.

**Fig. 6:**
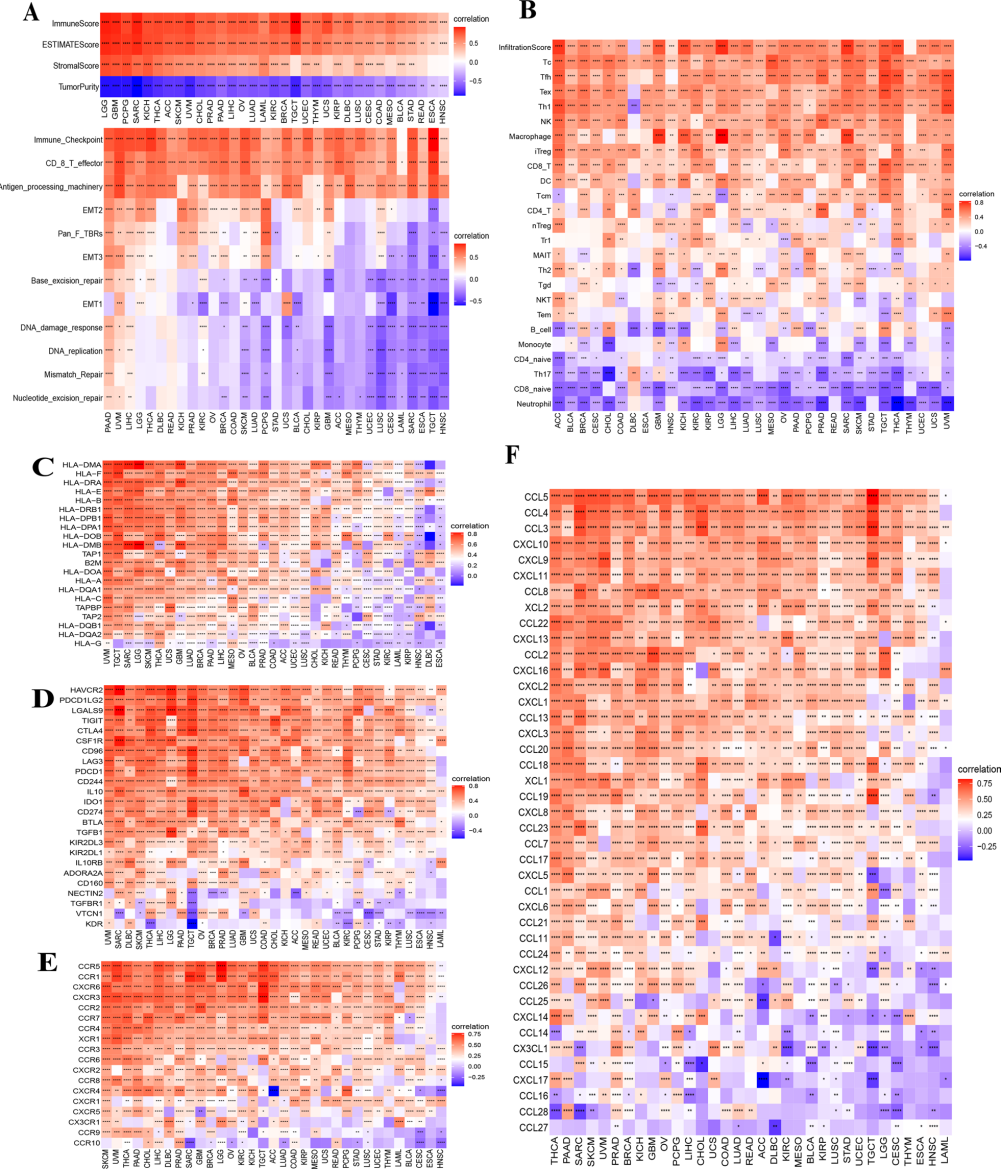
Correlations between pyroptosis level and immune features. **A** Immune. Microenvironment. **B** Immune cell subsets. **C** Major histocompatibility complex-related genes. **D** Immune suppression genes. **E–D** Chemokines and their receptors

Considering the important function of the immune response process in tumorigenesis, we explored the correlation of pyroptosis with the immune microenvironment in cancers. The results showed that the immune score and stromal score were positively associated with the pyroptosis score, while the pyroptosis score was negatively associated with tumour purity (Fig. 6A). Furthermore, we investigated the associations between the pyroptosis score and immune-related pathways, matrix/metastasis-related pathways, and DNA damage repair pathways. The results showed that the pyroptosis score was positively associated with the immune checkpoint, CD_8_T effector, and antigen processing machinery pathways in almost all cancers. DNA damage repair was negatively associated with the pyroptosis score in most types of cancers, especially HNSC, TGCT, ESCA, SARC, LAML, CEUS, GBM, and PCPG. The pyroptosis score was positively associated with EMT2 in cancers, with pan_F TBRs in 14 cancers, and with EMT3 in 16 cancers, while EMT1 showed a positive correlation with pyroptosis in 11 cancers (Fig. 6A). To better understand the correlations of pyroptosis with immunotherapy, we calculated the Spearman correlation coefficients of the pyroptosis score and immune cell infiltration and found that the pyroptosis score was positively associated with the immune cell infiltration score in almost all cancers except THYM and DLBC (Fig. 6B). The pyroptosis score was positively correlated with most T cells, such as Tc, Tfh, Tex, Th1, iTreg, CD8_T, CD4_T, and Tr1 cells, in most types of cancers. Positive associations were observed between the pyroptosis score and macrophages, DCs, NK cells, and T cells in most cancers (Additional file 2: Fig. S8A-B). In contrast, the pyroptosis score showed negative associations with naïve CD8 T cells, neutrophils, and Th17 cells. Some immune cells had individual patterns. For example, Th1 and Th2 cells were only negatively associated with pyroptosis levels, while Th17 cells showed a positive association with the pyroptosis level in DLBC.

We also investigated the associations between the pyroptosis level and MHC genes (Fig. 6C), immunosuppressive genes (Fig. 6D), chemokines (Fig. 6E) and their receptors (Fig. 6F). The results showed that MHC genes, immunosuppressive genes, chemokines and their receptors were positively associated with the pyroptosis level in most types of cancers. Pyroptosis was negatively correlated with these immune-related genes in some cancers. For example, human leukocyte antigen (HLA-DMA), HLA-DOB, and HLA-DQB1 were negatively associated with pyroptosis in DLBC, and KDR (an immune suppressor gene) was negatively associated with the pyroptosis level in TGCT. Similarly, chemokines (CXCR4) showed a negative association with pyroptosis in ACC. Some chemokine receptors, such as cCCL27 (8 cancers), CCL28 (7 cancers), CCL16 (5 cancers), CCL17 (5 cancers), and CCL15 (four cancers), were negatively associated with pyroptosis in several cancers. CCL11 showed a negative relationship with the pyroptosis level in only DLBC. These results indicated a close association between pyroptosis and the immune microenvironment in cancers, but more research is required to fully elucidate the details.

We further evaluated the correlations of the pyroptosis level with microsatellite instability (MSI) and the tumour mutation burden (TMB), which were suggested to be associated with the prognosis of multiple cancers after immunotherapy. We observed that the pyroptosis level was positively associated with MSI in COAD, STAD, THCA, and PRAD, while negative relationships were observed in LIHC, KIRP, OV, PADD, TGCT and DLBC (Additional file 2: Fig. S9A). For TMB, the pyroptosis level showed a positive association in COAD and STAD but a negative association in LUAD, PCPG, TGCT and CHOL (Additional file 2: Fig. S9B).

**Fig.7:**
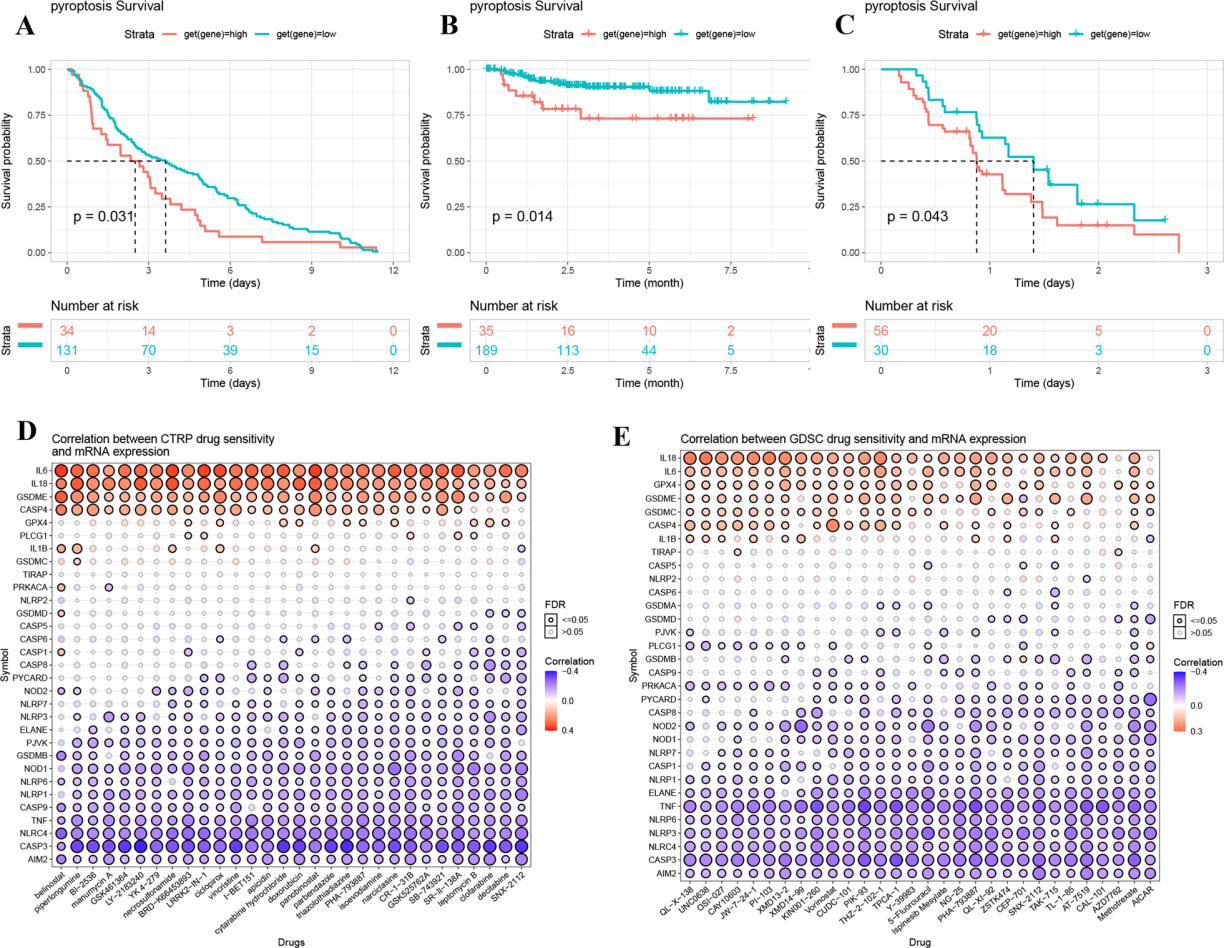
Associations between pyroptosis and immunotherapy and drug sensitivity. **A–C** Effect of pyroptosis on survival outcomes in primary bladder cancer, urothelial carcinoma, and non-squamous non-small cell lung cancer after immunotherapy. **D**, **E** Correlation between drug sensitivity of CTRP, GDSC and pyroptosis genes expression

To better understand the correlation of pyroptosis with immunotherapy, we investigated the effect of the pyroptosis level on prognosis using three GEO cancer datasets (GSE13507: primary bladder cancer; GSE32894: urothelial carcinoma; GSE61676: non-squamous non-small cell lung cancer). The results showed that a high pyroptosis level was associated with poor OS in primary bladder cancer (Fig. 7A), with DFS in urothelial carcinoma (Fig. 7B), and with OS in non-squamous non-small cell lung cancer after immunotherapy (Fig. 7C). These results implied that pyroptosis might affect immunotherapeutic efficacy in some cancers.

### Identification of potential compounds targeting pyroptosis-related genes

To further understand the association between pyroptosis and drug sensitivity, we calculated the correlation coefficients between pyroptosis genes and drug sensitivity (evaluated by the percent viability curve approach) using the CTRP and GDSC datasets. We selected the top 30 compounds targeting pyroptosis-related genes (|r|> 0.3). The results showed that the expression levels of IL-6, IL18 and GSDME were positively associated with these compounds, while CASP4 was positively correlated with sensitivity to 26 cancer drugs (Fig. 7D). AIM2, CASP3, NLRC4, TNF, and NLRP6 may be associated with tumour drug resistance (Fig. 7E). The other results for the two datasets are presented in Additional file 1: Table S6 and Additional file 1: Table S7. These results indicated that pyroptosis might be associated with cancer sensitivity to multiple drugs.

## Discussion

Pyroptosis is an inflammatory programmed cell death method that is characterized by the activation of inflammatory caspases (caspases 1, 4, 5 and 11) in inflammasomes and the secretion of inflammatory cytokines such as interleukin-1β and interleukin-18 [20]. Researchers have found that GSDMA acts as an essential downstream substrate of inflammatory caspases, inducing pyroptosis by forming pores in the plasma membrane [21]. Currently, the functions of pyroptosis in tumorigenesis and tumour treatment are being increasingly studied. However, compressive analysis and an understanding of the biological regulation of pyroptosis genes are lacking in cancers. In the present study, we integrated multiomics data and clinically relevant outcomes across 33 cancers from a public dataset and depicted the landscape of alterations and epigenetic and transcript levels of pyroptosis genes. We also evaluated the pyroptosis levels across cancers using ssGSEA and identified the correlations between the pyroptosis level and immune features, survival outcomes, immunotherapy, and drug sensitivity. Pyroptosis showed different genetic, epigenetic, and transcriptional patterns in different cancers, and differential effects of pyroptosis on survival and immune treatment were observed, especially in KIRC, LGG, GBM, PADD, and SKCM. An increased pyroptosis level has an adverse effect on the immunotherapeutic treatment of primary bladder cancer, urothelial carcinoma, and non-squamous non-small cell lung cancer, which means that the pyroptosis level should be considered in cancer immunotherapies.

The molecular mechanism of pyroptosis in tumorigenesis and development remains unclear. However, the correlations of pyroptosis with some functions and pathways may provide some important clues. The GSVA results indicated that the pyroptosis genes were mainly enriched in two components. The first was inflammation-related pathways, such as the IL6/JAK/STAT3 signalling, inflammatory response, IL2/STAT5 signalling, TNF-alpha signalling via NFKB, and KRAS signalling pathways, which have been proven to be associated with tumours. The inflammatory response can promote the occurrence and progression of tumours [22]. Sustained oxidative stress during the process of chronic inflammation leads to DNA damage and inhibition of DNA damage repair, resulting in inactivation of tumour suppressor genes. Inflammatory cells and inflammatory factors in the microenvironment can induce the expression of a variety of cytokines [23]. These inflammatory cells, cytokines and their downstream products promote the occurrence, development, and metastasis of cancer via various mechanisms, such as by inhibiting apoptosis, promoting angiogenesis and inducing immune tolerance [24]. The inflammatory response is the primary characteristic of pyroptosis. The pyroptosis process depends on the activation of Caspase-1, which importantly functions to mediate the cleavage of the interleukin-1 β precursor into active IL-1β. IL-1β can recruit and activate other immune cells and induce the synthesis of chemokines, inflammatory factors, adhesion molecules, etc., eventually causing the cascade effect, which amplifies the inflammatory response and leads to a severe inflammatory response [25]. We also found that pyroptosis genes were enriched in immune-related pathways such as the allograft rejection, complement, and interferon alpha and gamma response pathways. The GSEA results indicated that several immune-related pathways, such as the innate immune system, adaptive immune system, and cytokine signalling in the immune system, were associated with multiple cancers. Pyroptosis, as a form of programmed cell death, is an important natural immune response of the body. A previous study reported that the induction of pyroptosis in tumours induced high antitumour immune activity and promoted tumour clearance [26, 27]. However, our results indicated that pyroptosis had a favourable effect on survival outcomes in BRCA, KICH, MESO, SARC, SKCM, STAD, TCHA and BLCA, while pyroptosis exerted adverse prognostic effects in ESCA, GBM, HNSC, KIRC, LAML, LGG, LUSC, PADD, THYM, UCES, UCS and UVM. Previous studies have also reported that pyroptosis inhibits the progression of breast, liver, ovarian, stomach, and colon cancers and promotes the progression of melanoma. NLRP3 inflammasome-mediated pyroptosis promotes the progression of lung adenocarcinoma but inhibits the progression of non-small-cell lung cancer, while GSDMD-mediated pyroptosis promotes the progression of non-small-cell lung cancer [28]. Our results validated the dual roles of pyroptosis in cancers. In addition, tumours at the same location, such as the thyroid, kidneys, and lungs, with the same pyroptosis level have different prognostic patterns. Furthermore, we analysed the survival outcomes of patients subjected to immunotherapy and found that elevated pyroptosis levels were correlated with an adverse prognosis in primary bladder cancer, urothelial carcinoma, and non-squamous non-small-cell lung cancer. Pyroptosis does not always exert antitumour immune effects.

We further analysed the correlation between drug sensitivity and pyroptosis gene expression. A previous study reported that GSDMD is activated by the cleavage of Caspase-3 and induces pyroptosis in response to tumour chemotherapy drugs [29]. Human neuroblastoma SH-SY5Y cells and human malignant melanoma MeWo cells have high GSDME expression levels. Under the action of chemotherapy drugs such as topotecan, etoposide, and cisplatin, the cells undergo obvious pyroptosis rather than apoptosis [30, 31]. Our results indicated that GSDME was positively associated with multiple molecular drugs. GSDME, as a tumour suppressor gene, is expected to be the target of a new clinical treatment direction. NLRP1 expression showed a negative association with drug sensitivity, proving its tumour promotional role. A previous study reported that NLRP1 promotes melanoma growth by enhancing inflammasome activation and suppressing apoptotic pathways [12]. These results highlight the dual roles of pyroptosis in cancers.

Our study has several advantages over previous studies on certain cancers. First, previous studies focused on the development and validation of prognostic models involving certain cancer types [32–35]. Our studies aimed to assess pyroptosis levels in cancer as a whole, and our model of pyroptosis levels is thus more universal. Second, previous studies tended to assess only one survival outcome (usually OS), and our study assessed four survival outcomes (OS, DSS, PFI, and DFI) in all kinds of cancers. Third, we first assessed the association of the pyroptosis level with immunotherapeutic responses based on available data, and this association has never been reported for certain cancers. Finally, our results depicted a landscape of gene dysregulation, signalling pathways, immune therapy, model assessment, and clinical relevance in cancers, which has important significance for guidelines and practice.

Some study limitations should be addressed. The effect of the pyroptosis level on immunotherapy in several cancers was not assessed due to a lack of available immunotherapy data. In addition, this study did not explore the molecular mechanisms of pyroptosis in cancers. Future studies should validate the effect of the pyroptosis level on immunotherapy in more cancers, and the potential molecular mechanisms should also be explored to identify potential treatment targets in certain cancers.

## Conclusions

In the present study, we performed a comprehensive analysis of pyroptosis and genes regulating pyroptosis in cancers. Our study found that (1) aberrantly expressed pyroptosis genes are mainly attributed to CAN frequency and differences in DNA methylation levels in cancer. (2) Moreover, the established pyroptosis level model based on the ssGSEA method uncovers the dual roles of pyroptosis in different cancers, and (3) the pyroptosis level is associated with clinical prognosis in multiple cancers, especially LGG, GBM, KIRC, PAAD, SKCM, UVM, BLCA, COAD, THYM, and SARC. (4) The dual role of pyroptosis also affects the immunotherapeutic efficacy in several cancers, including bladder cancer, urothelial carcinoma, and non-squamous non-small cell lung cancer, and (5) six pyroptosis genes (AIM2, CASP3, CASP4, NLRC4, NLRP6, and TNF) are closely correlated with drug sensitivity across cancers and may be considered therapeutic targets in cancer. Our comprehensive analysis highlighted the possibility of pyroptosis-based cancer therapeutic strategies.

## Supplementary Information

**Additional file 1: Table S1.** Lists of cancer types. **Table S2.** The 33 pyroptosis-associated genes used for classification. **Table S3.** Association between pyroptosis genes mutation and prognosis among cancers. **Table S4.** Methylation differences for pyroptosis genes in cancers. **Table S5.** Correlation of DNA methylation with pyroptosis genes expression. **Table S6.** CTRP drug sensitivity for pyroptosis genes in cancers. **Table S7.** GDSC drug sensitivity for pyroptosis genes in cancers.

**Additional file 2: Fig. S1.** Protein–protein interaction network of pyroptosis genes. **Fig. S2.** Gene alteration of pyroptosis genes in each cancer. **A:** heterozygous copy number variation in each cancer. **B:** homozygous copy number variation in each cancer **C:** The gene mutation frequency of pyroptosis genes in overall cancer. **Fig. S3.** Heatmap showed the mutation frequency of pyroptosis in each cancer. **Fig. S4.** Aberrant expression of pyroptosis-related genes among cancers. **Fig. S5.** Pyroptosis and survival prognosis based on Kaplan–Meier analysis. A: elevated pyroptosis level favors survival in ESCA, GBM, HNSC, KIRC, LAML, LGG, LUSC, PAAD, THYM, UCES, UCS, UVM. **B:** Elevated proptosis level disfavors survival in BRCA, KICH, MESO, SARC, SKCM, STAD, THCA, BLCA. **Fig. S6.** Risky score of pyroptosis genes in each cancer. **Fig. S7.** KEGG pathway enrichment of pyroptosis in several cancer. **A–F:** BRCA, GBM, HNSC, KIRC, LUSC, PAAD. **Fig. S8.** Correlations of pyroptosis level with immune cells infiltration. **A:** B cell, T cell, myeoid dendritic cell, endothelial cell. **B:** immune cell cibersort. **Fig. S9.** Correlations of pyroptosis level with microsatellite Instability **(A)** and tumor mutation burden **(B)**.

## Data Availability

The data underlying this article will be shared on reasonable request to the corresponding author.

## Abbreviations

GSDMD: Gasdermin D
NLRP: Nucleotide-Binding Oligomerization Domain, Leucine Rich Repeat and CARD Domain Containing
IL: Interleukin
NSCLC: Non-small cell lung carcinoma
UCSC: University of California SANTA CRUZ
GEO: Group on Earth Observations
RIG: Retinoic acid-induced gene
TCGA: The cancer genome atlas
GDSC: Genomics of Drug Sensitivity in Cancer: GSVA: Gene set variation analysis
FC: Fold change
DEG: Differentially expressed gene
GSEA: Gene set enrichment analysis
TME: Tumour microenvironment
DNA: Deoxyribonucleic acid
lncRNA: Long non-coding ribose nucleic acid
CNV: Copy number variation
TNF: Tumour necrosis
NF-kB: Nuclear factor kappa B
EMT: Epithelial-mesenchymal transition
HR: Hazard ratio
CI: Confidence interval
OS: Overall survival
DSS: Disease specific survival
PFI: Progression free interval
DFI: Disease-free interval
Tem: Effective memory T cell
Th1: T helper cell 1
Th17: T helper cell 17
Th2: T helper cell 2
Treg: Regulatory T cells
HAL: Human leukocyte antigen
TMB: Tumour mutation burden
