# Supplementary figures and images for: Pan-cancer analyses of pyroptosis with functional implications for prognosis and immunotherapy in cancer
Source: J Transl Med. 2022 Mar 4;20:109. doi: 10.1186/s12967-022-03313-x (PMC8896277; doi:10.1186/s12967-022-03313-x)

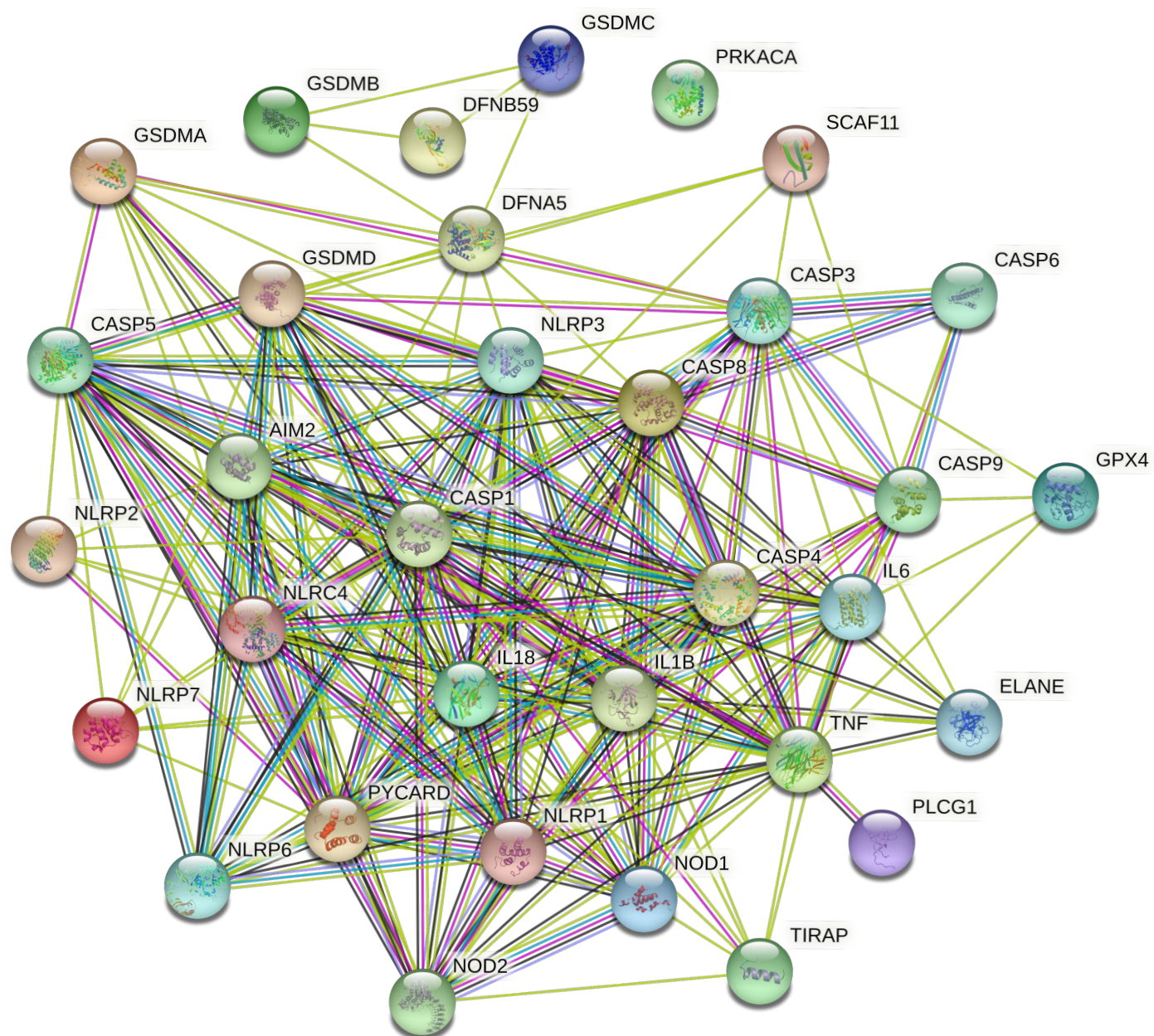

**Figure S1**

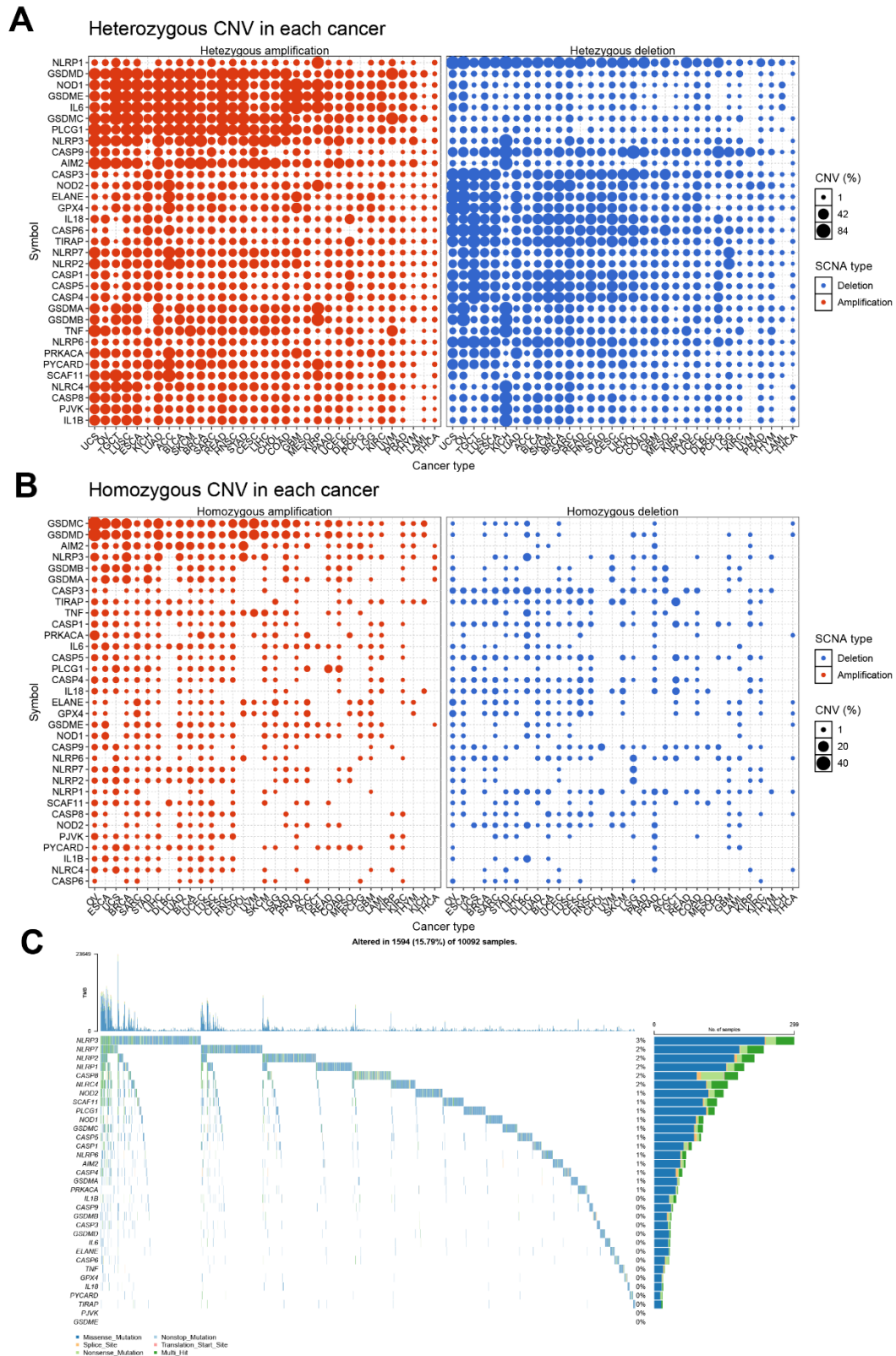

**Figure S2**

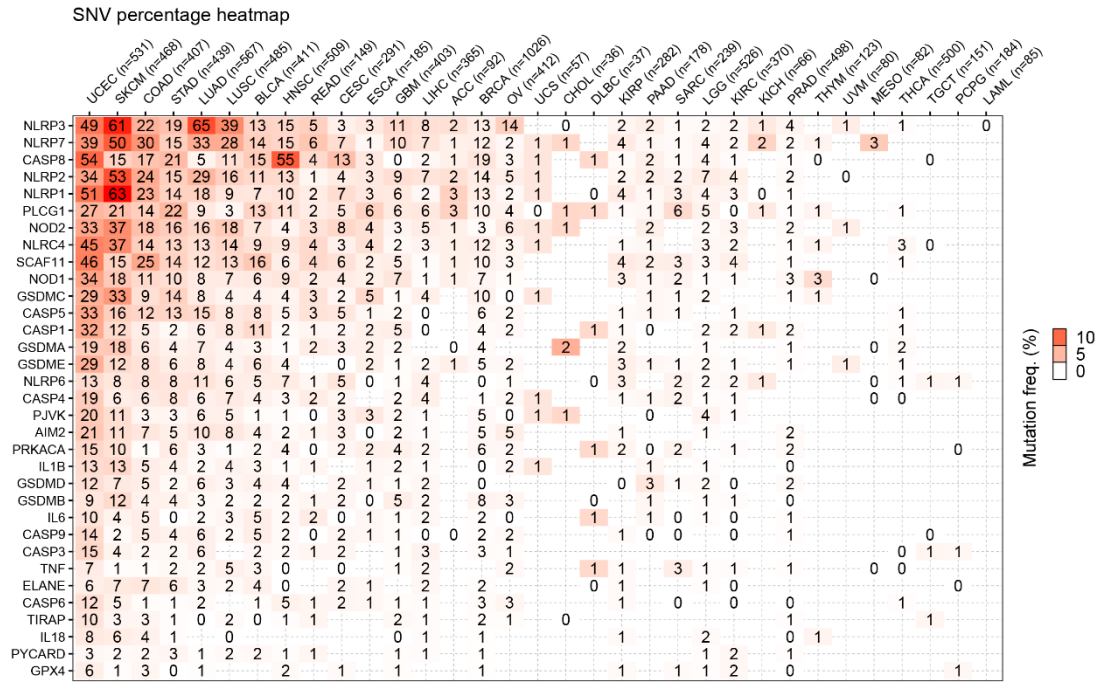

Figure S3

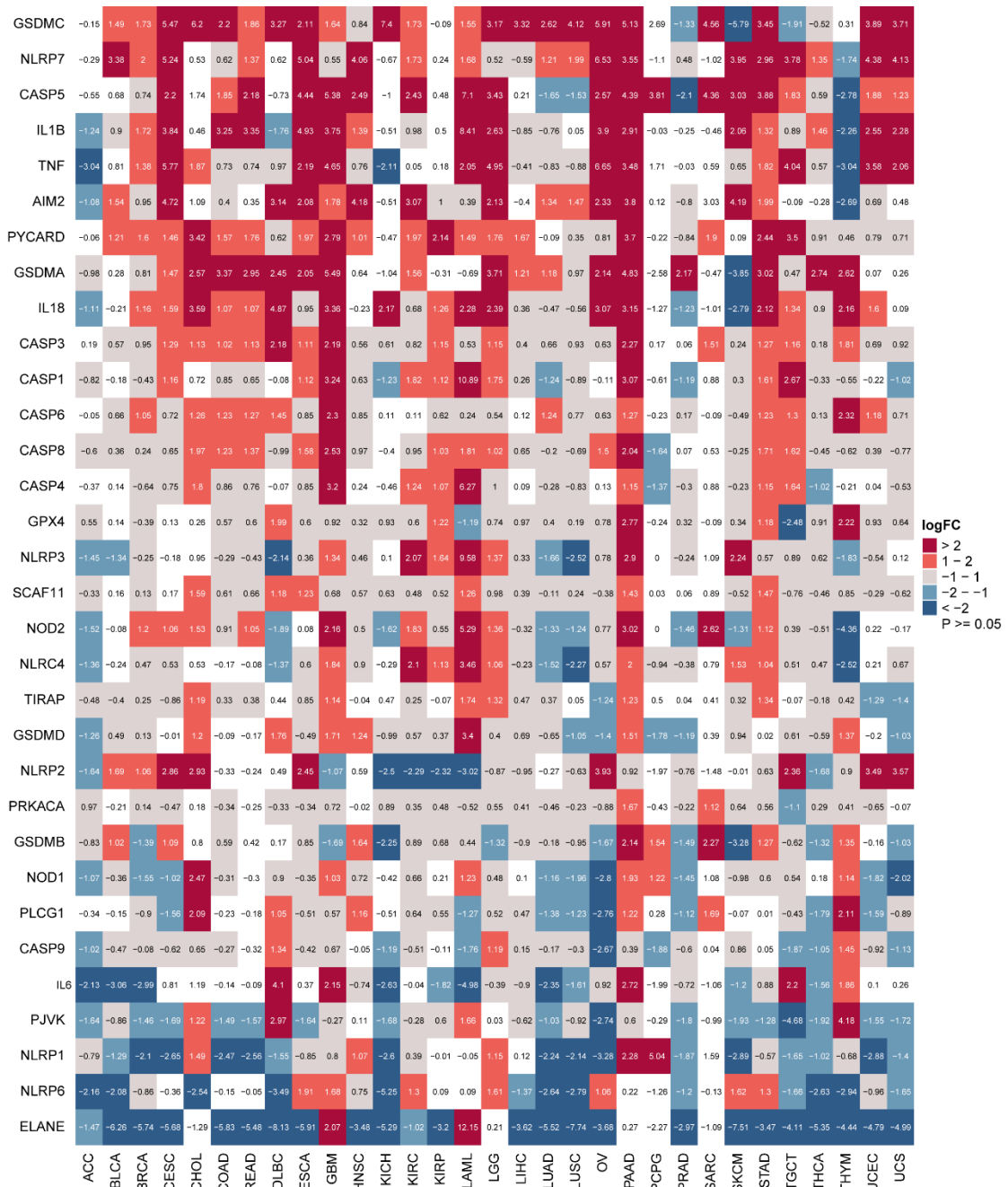

Figure S4

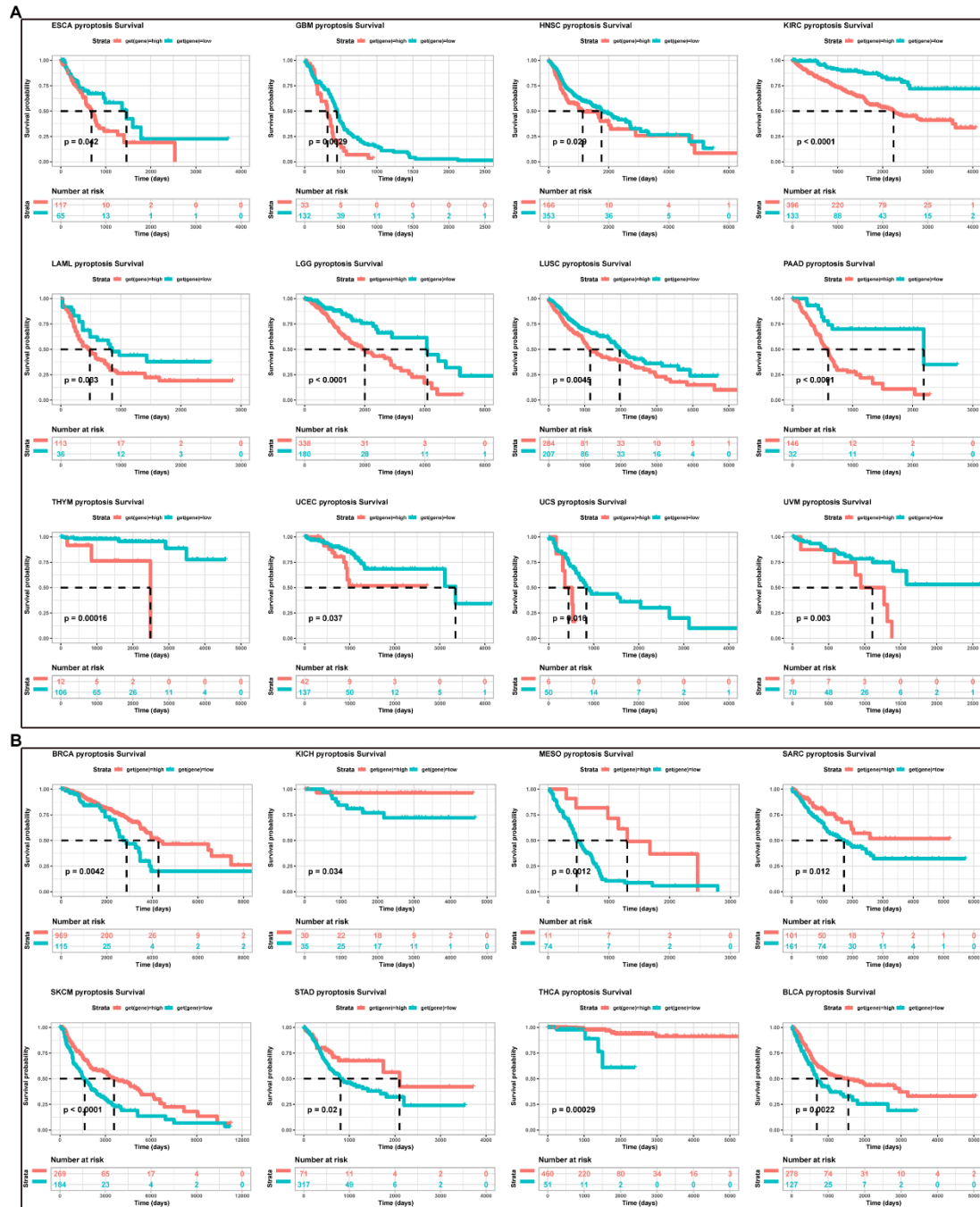

**Figure S5**

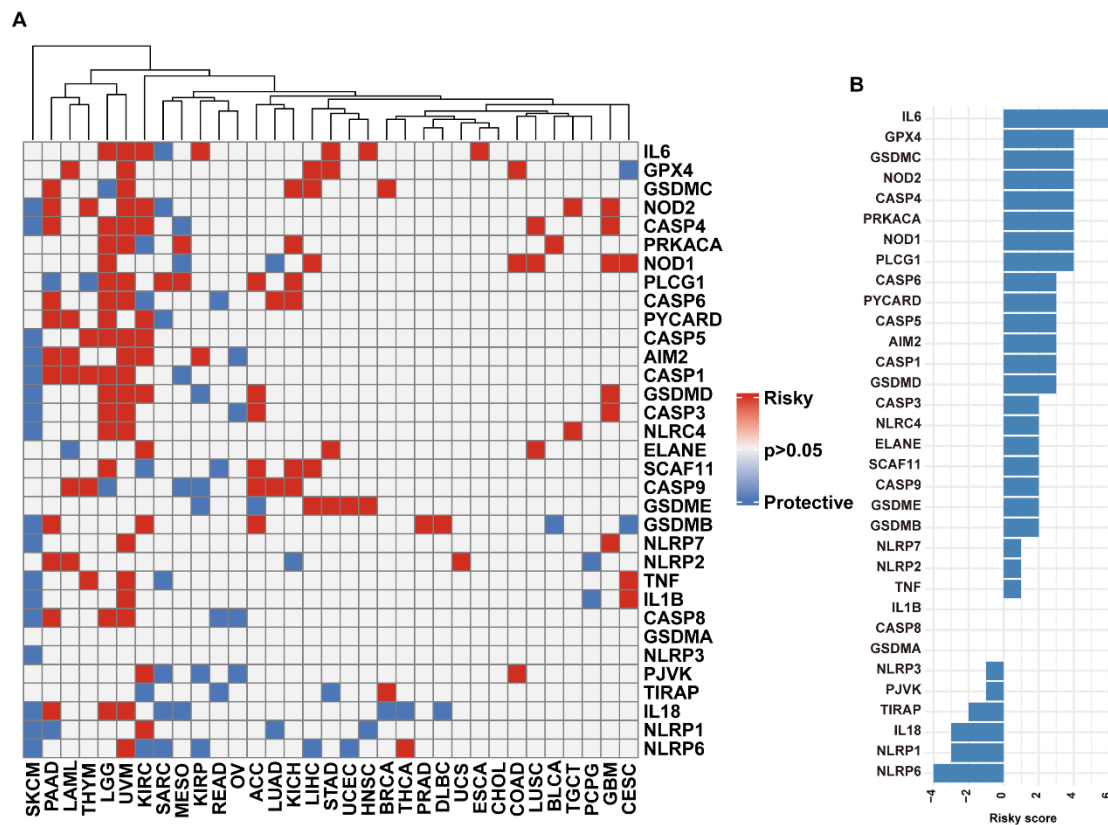

**Figure S6**

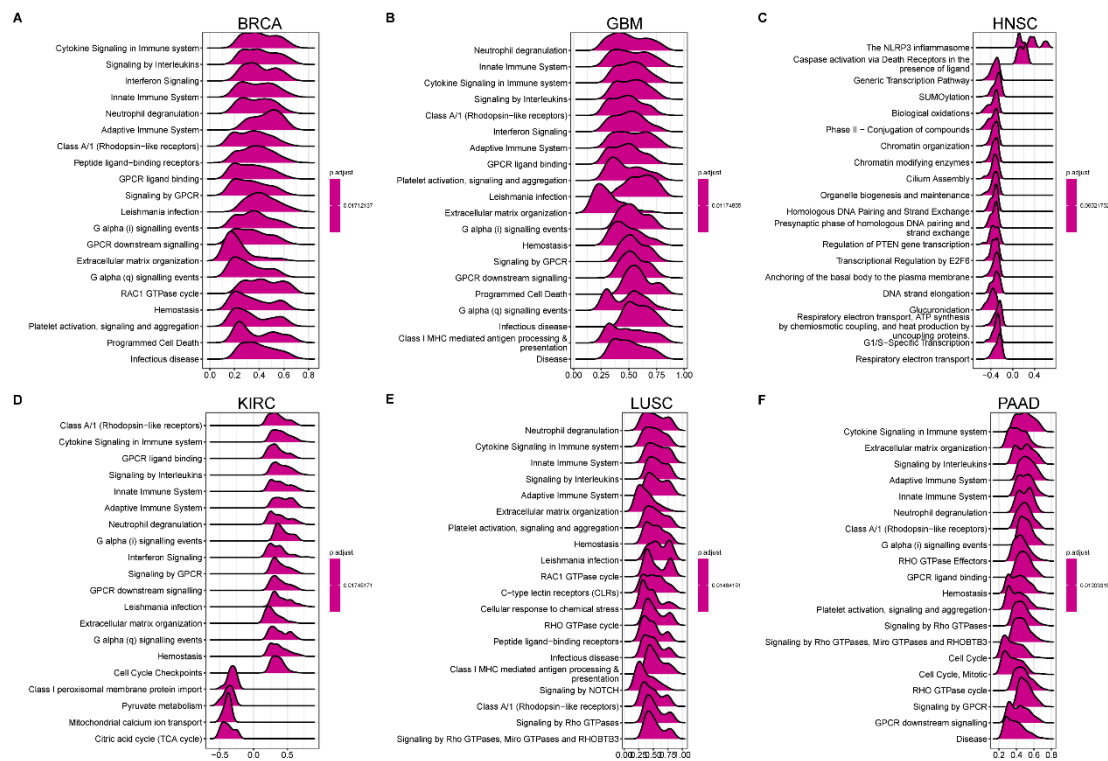

**Figure S7**

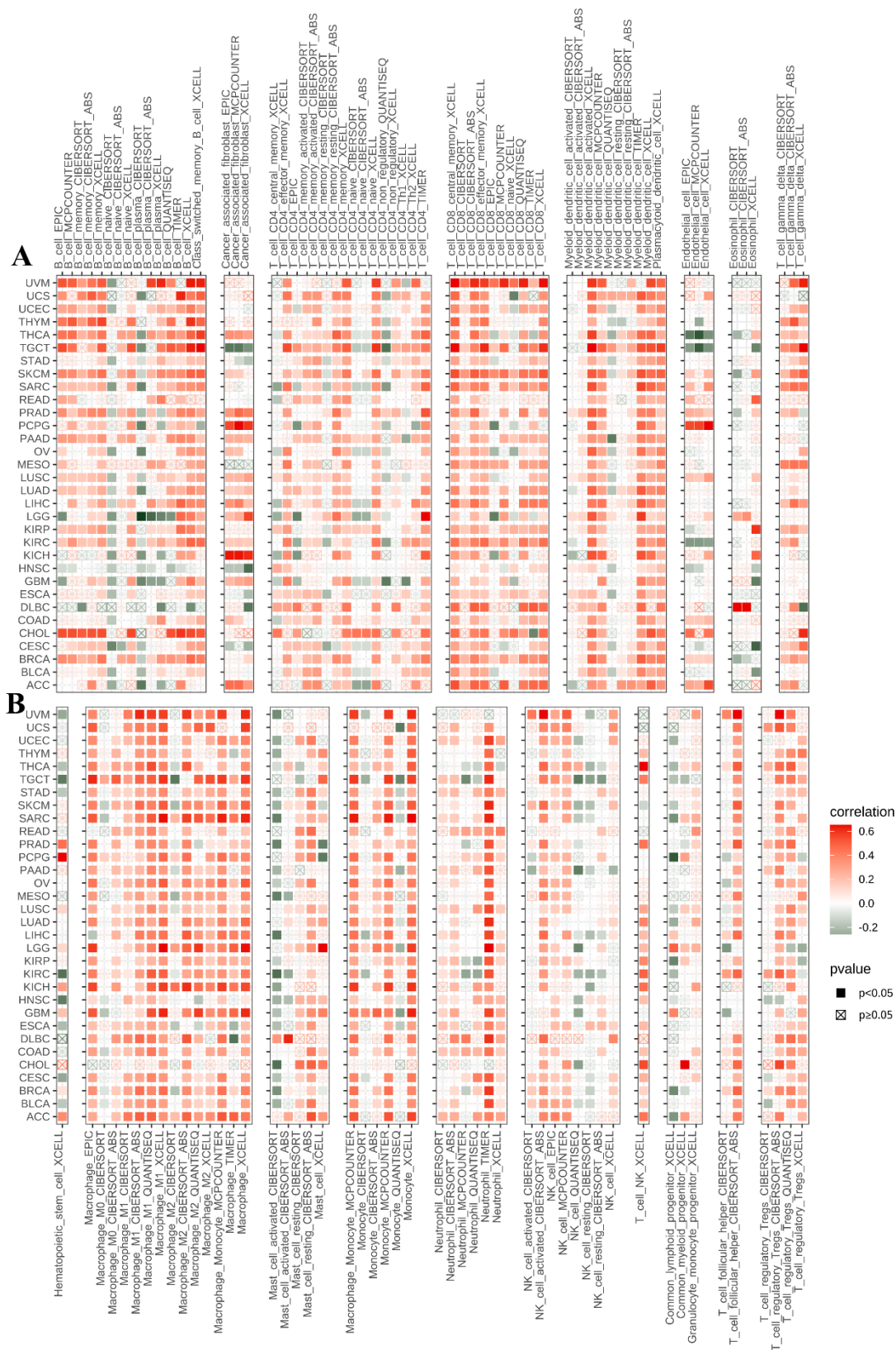

**Figure S8**

**A**

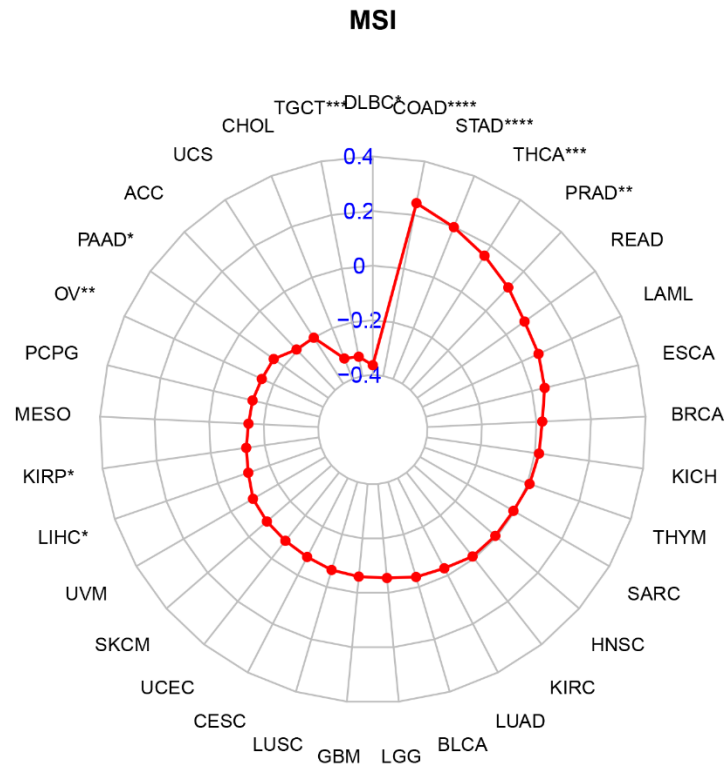

**B**

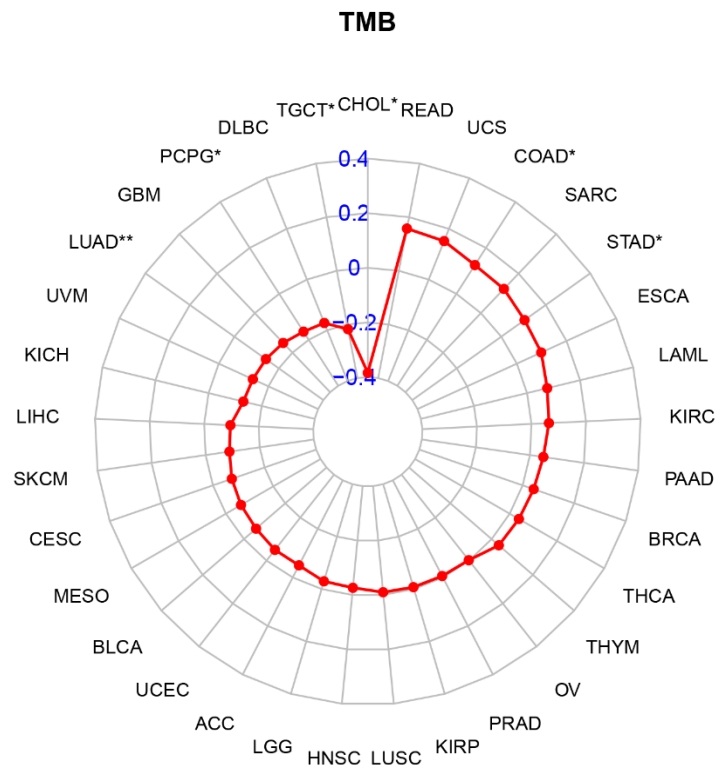

**Figure S9**

Supplement: Supplementary file 2 — Additional file 2: Fig. S1. Protein–protein interaction network of pyroptosis genes. Fig. S2. Gene alteration of pyroptosis genes in each cancer. A: heterozygous copy number variation in each cancer. B: homozygous copy number variation in each cancer C: The gene mutation frequency of pyroptosis genes in overall cancer. Fig. S3. Heatmap showed the mutation frequency of pyroptosis in each cancer. Fig. S4. Aberrant expression of pyroptosis-related genes among cancers. Fig. S5. Pyroptosis and survival prognosis based on Kaplan–Meier analysis. A: elevated pyroptosis level favors survival in ESCA, GBM, HNSC, KIRC, LAML, LGG, LUSC, PAAD, THYM, UCES, UCS, UVM. B: Elevated proptosis level disfavors survival in BRCA, KICH, MESO, SARC, SKCM, STAD, THCA, BLCA. Fig. S6. Risky score of pyroptosis genes in each cancer. Fig. S7. KEGG pathway enrichment of pyroptosis in several cancer. A–F: BRCA, GBM, HNSC, KIRC, LUSC, PAAD. Fig. S8. Correlations of pyroptosis level with immune cells infiltration. A: B cell, T cell, myeoid dendritic cell, endothelial cell. B: immune cell cibersort. Fig. S9. Correlations of pyroptosis level with microsatellite Instability (A) and tumor mutation burden (B). [file 12967_2022_3313_MOESM2_ESM.pdf]
